# Fetal overgrowth in women with type 1 and type 2 diabetes mellitus

**DOI:** 10.1371/journal.pone.0187917

**Published:** 2017-11-09

**Authors:** Linnea Ladfors, Nael Shaat, Nana Wiberg, Anastasia Katasarou, Kerstin Berntorp, Karl Kristensen

**Affiliations:** 1 Department of Obstetrics and Gynecology, Skåne University Hospital, Malmö, Sweden; 2 Department of Endocrinology, Skåne University Hospital, Malmö, Sweden; 3 Department of Clinical Sciences, Lund University, Malmö, Sweden; 4 Department of Clinical Sciences, Lund University, Lund, Sweden; Centro Cardiologico Monzino, ITALY

## Abstract

**Objectives:**

Despite improved glycemic control, the rate of large-for-gestational-age (LGA) infants remains high in pregnancies complicated by diabetes mellitus type 1 (T1DM) and type 2 (T2DM). Poor glycemic control, obesity, and excessive gestational weight gain are the main risk factors. The aim of this study was to determine the relative contribution of these risk factors for LGA in women with T1DM and T2DM, after controlling for important confounders such as age, smoking, and parity.

**Methods:**

In this retrospective chart review study, we analyzed the medical files of pregnant women with T1DM and T2DM who attended the antenatal care program at Skåne University Hospital during the years 2006 to 2016. HbA1c was used as a measure of glycemic control. Maternal weight in early pregnancy and at term was registered. LGA was defined as birth weight > 2 standard deviations of the mean. Univariable and multivariable logistic regression analysis was used to calculate odds ratios (OR’s) and 95% confidence intervals (CIs) for LGA.

**Results:**

Over the 11-year period, we identified 308 singleton pregnancies in 221 women with T1DM and in 87 women with T2DM. The rate of LGA was 50% in women with T1DM and 23% in women with T2DM. The multivariable regression model identified gestational weight gain and second-trimester HbA1c as risk factors for LGA in T1DM pregnancies (OR = 1.107, 95% CI: 1.044–1.17, and OR = 1.047, 95% CI: 1.015–1.080, respectively) and gestational weight gain as a risk factor in T2DM pregnancies (OR = 1.175, 95% CI: 1.048–1.318), independent of body mass index.

**Conclusions:**

Gestational weight gain was associated with LGA in women with T1DM and T2DM, independent of maternal body mass index. The findings suggest that monitoring and regulation of gestational weight gain is important in the clinical care of these women, to minimize the risk of fetal overgrowth.

## Introduction

Due to improved antenatal and intrapartum care, the outcomes in pregnant women with type 1 and type 2 diabetes mellitus (T1DM and T2DM) have improved in recent decades. However, the prevalence of fetal overgrowth (macrosomia and large-for-gestational-age (LGA) infants) remains high, resulting in an increased risk of maternal and neonatal complications [1, 2]. The maternal complications include prolonged labor, third- and fourth-degree perineal tears, postpartum hemorrhage, and cesarean section [3–5]. The neonatal complications include hypoglycemia, respiratory disorders, hyperbilirubinemia, and shoulder dystocia [6].

Some adverse consequences of being LGA may persist into later life. There are study results which suggest that being born LGA predispose to overweight and obesity in childhood and adolescence, and also to subsequent comorbidities such as T2DM and cardiovascular disease [7, 8].

The fetal overgrowth is believed to be caused mainly by the placental transfer of maternal glucose, leading to fetal hyperinsulinemia, which drives fetal fat accumulation [9]. However, in spite of improved glycemic control in pregnant women with diabetes, the risk of fetal overgrowth remains high, suggesting that other contributory factors may be involved. The contribution of obesity and gestational weight gain (GWG) to LGA and macrosomia has been highlighted in several studies [10–13]. The body mass index- (BMI-) adjusted recommendations for GWG were issued by the Institute of Medicine (IOM) with the intention of reducing the risk of fetal overgrowth and associated complications [14]. Since the underlying pathophysiology of T1DM and T2DM is fundamentally different, it is reasonable to assume that factors known to affect fetal growth may have different roles in T1DM and T2DM pregnancies.

The aim of this study was to determine the relative contribution of the major risk factors for LGA and macrosomia, and to evaluate the BMI-adjusted recommendations for GWG in women with T1DM and T2DM.

## Methods

### Study design and study subjects

This retrospective chart review study included a total of 308 women with pre-gestational T1DM and T2DM who attended antenatal care and gave birth at the Department of Obstetrics and Gynecology, Skåne University Hospital (in Lund and Malmö) between 2006 and 2016. We included singleton pregnancies for which medical records for antenatal care and delivery were available. Women with multiple gestations, miscarriages, or intrauterine fetal deaths were not included. Pregnancies complicated by chromosomal disorders, major fetal anomalies, or syndromes were excluded.

The T2DM pregnancies were managed according to our routine procedures for T1DM pregnancies. Prior to 2015, treatment with oral antidiabetic drugs was discontinued at the first antenatal visit and replaced with insulin. From 2015, metformin was an accepted treatment option. If glucose targets were not achieved on diet and metformin alone, additional treatment with insulin was given. In all, 7 of 87 women with T2DM continued with metformin.

### Variables

The following data were retrieved from the medical files: date of birth; ethnicity; date of delivery; HbA1c levels in the first trimester (weeks 10‒14); in the second trimester (weeks 22‒26), and in the third trimester (weeks 32‒36); type of diabetes and treatment; parity; weight and height at the initial visit; smoking habits; weight at term; gestational age at delivery; mode of delivery; birth weight; gender of offspring; and Apgar score at 5 minutes. Maternal complications such as pre-eclampsia and pregnancy-induced hypertension were recorded, as were neonatal complications such as hypoglycemia, jaundice, and shoulder dystocia. The maternal BMI was calculated at the pre-pregnancy visit or at the first visit to the antenatal clinic at 6–8 weeks. All pregnancies were dated by ultrasound in the first trimester.

HbA1c was measured with ion-exchange chromatography. Values given in % (Mono S) were converted to IFCC units (mmol/mol) using the regression equations developed by the IFCC Working Group [15].

LGA was defined as birth weight > 2 standard deviations (SD’s) of the expected birth weight for gestational age according to the Swedish standard for intrauterine growth [16]. Macrosomia was defined as birth weight > 4,000 g [17].

Excessive GWG was defined as weight gain exceeding the IOM recommendations for maternal weight gain during pregnancy, after adjustment for the maternal pre-pregnancy BMI. For women with normal weight (BMI 18.5–24.9), a weight gain of 11.3–15.9 kg is recommended. For those who are overweight (BMI 25–29.9), a weight gain of 6.8–11.3 kg is recommended; and for women who are obese (BMI ≥ 30), a weight gain of 5–9.1 kg is recommended [14].

### Statistical analysis

The t-test for independent samples was used to compare normally distributed continuous variables, and the Mann-Whitney *U*-test was used to compare data that were not normally distributed. The Chi-squared test was used to compare categorical variables. Logistic regression was used to analyze the effect of the clinical predictors on LGA. Univariable binary regression was used for each variable followed by a multivariable logistic regression model where all interesting variables were retained while controlling for important confounders such as age (continuous variable), smoking (yes/no), and parity (1/> 1). Odds ratios (OR’s) and 95% confidence intervals (CIs) were calculated. Two-sided *p*-values of less than 0.05 were considered to be statistically significant. IBM SPSS Statistics version 24.0 for Windows (IBM Corporation, Armonk, NY) was used for analysis.

### Ethics

The study was approved by the Ethics Committee of Lund University (LU 48–2012) and was conducted in accordance with the Swedish Act on Ethics Review of Research Involving Humans (2003:460) and the Swedish Act on Personal Data (2008:355), revised 2009 (2009:525). The Ethics committee advised that no informed consent was deemed necessary for this study as data was used for quality control and collected as part of routine clinical care.

## Results

The cohort consisted of 221 women with T1DM and 87 women with T2DM. The maternal characteristics are shown in Table 1. The women with T1DM were generally younger and leaner than the women with T2DM. The women with T1DM gained significantly more weight during pregnancy, exceeding the recommendations by IOM in 51% of the cases. They also had higher HbA1c levels in all three trimesters compared to the women with T2DM. A higher proportion of women with T2DM were of Non-Nordic origin (with women from the Middle East, Africa, and Asia comprising the largest groups).

**Table 1:** Maternal characteristics.

	T1DM (n = 221)	T2DM (n = 87)	*p*-value
Age, years	31.9 ± 5.0	34.7 ± 4.9	0.000
Smokers	25 (11)	11 (13)	0.742
Nordic-Caucasian	192 (87%)	57 (65%)	0.000
Primipara	99 (45)	16 (18)	0.000
BMI in early pregnancy, kg/m^2^	24.6 (22.7–28.1)	31.1 (27.1–36.1)	0.000
Gestational weight gain, kg	13 (10–17)	10 (6–13)	0.000
Excessive weight gain	112 (51)	32 (37)	0.000
HbA1c trimester 1, mmol/mol	50 ± 13	44 ± 13	0.002
HbA1c trimester 2, mmol/mol	39 ± 12	36 ± 9	0.032
HbA1c trimester 3, mmol/mol	41 ± 10	36 ± 8	0.002

T1DM, type 1 diabetes mellitus; T2DM, type 2 diabetes mellitus; BMI, body mass index.

Results are given as n (%), mean ± SD, or median (interquartile range).

Differences in means were tested by t-test and differences in medians were tested by the Mann-Whitney *U*-test. Frequencies were compared using the Chi-squared test. Missing data were below 5% for all variables, except for weight gain (7%) and HbA1c (11% in trimester 1, 10% in trimester 2, and 29% in trimester 3).

Maternal and neonatal outcomes in women with T1DM and T2DM are presented in Table 2. Despite earlier delivery in general, the mean birth weight of the offspring was more than 200 g higher in T1DM pregnancies than in T2DM pregnancies. Similarly, the incidences of macrosomia, LGA, and neonatal hypoglycemia were significantly higher in T1DM offspring.

**Table 2:** Maternal and neonatal outcomes in T1DM and T2DM pregnancies.

	T1DM (n = 221)	T2DM (n = 87)	*p*-value
Pre-eclampsia/PIH	22 (10)	9 (10)	0.928
Cesarean section	92 (42)	30 (35)	0.237
Gestational age at delivery, weeks	37.1 ± 2.1	38 ± 2.2	0.000
Pre-term birth	63 (29)	11 (13)	0.003
Female infant	104 (47)	43 (49)	0.708
LGA infant	111 (50)	20 (23)	0.000
Birth weight, g	3746 ± 711	3531 ± 778	0.020
Macrosomia (> 4,000 g)	87 (39)	19 (22)	0.003
Apgar score < 7 at 5 min	9 (4)	5 (6)	0.536
Neonatal hypoglycemia	61 (28)	11 (13)	0.005
Shoulder dystocia	4 (2)	1 (1)	0.680

T1DM, type 1 diabetes mellitus; T2DM, type 2 diabetes mellitus; PIH, pregnancy-induced hypertension; LGA, large for gestational age.

Results are given as n (%) or mean ± SD.

Differences in means were tested by t-test. Frequencies were compared using the Chi-squared test.

The results of the univariable logistic regression analysis of variables tested for associations with LGA are presented in Table 3 (T1DM) and Table 4 (T2DM). In both groups of women, GWG per se (and also excessive GWG according to IOM guidelines) was significantly associated with fetal overgrowth (LGA). T1DM women with fetal overgrowth also had significantly higher levels of HbA1c in all three trimesters, but similar BMI in early pregnancy. In contrast, HbA1c levels were not associated with LGA in women with T2DM whereas BMI in early pregnancy was. Of the women with T1DM who gave birth to LGA infants, 50% were classified as having normal weight, 32% as overweight, and 18% as obese, as compared to 57%, 29%, and 14% in T1DM women who gave birth to non-LGA infants (not significant). The corresponding figures for women with T2DM were 10%, 25%, and 65% as opposed to 10%, 40%, and 49%, respectively (not significant).

**Table 3:** Results of univariable logistic regression analysis of variables tested for associations with LGA in women with T1DM.

	LGA (n = 111)	Non-LGA (n = 109)	OR	95% CI	*p*-value
Age at delivery, years	31.1 ± 5.1	30.7 ± 5.0	1.015	0.963–1.071	0.577
Smokers	12 (11)	13 (12)	0.904	0.392–2.082	0.812
Primipara	41 (37)	57 (52)	0.534	0.312–0.915	0.023
Weight in early pregnancy, kg	74 ± 12	72 ± 12	1.017	0.994–1.040	0.159
BMI in early pregnancy, kg/m^2^	25.8 ± 4.0	25.5 ± 3.7	1.021	0.952–1.094	0.562
Gestational weight gain, kg	15 ± 6	13 ± 5	1.091	1.034–1.151	***0*.*001***
Excessive weight gain	64 (58)	48 (44)	2.148	1.228–3.759	*0*.*007*
HbA1c trimester 1, mmol/mol	52 ± 13	48 ± 13	1.024	1.001–1.047	0.039
	(104)^a^	(101)^a^			
HbA1c trimester 2, mmol/mol	41 ± 12	37 ± 11	1.033	1.006–1.060	***0*.*015***
	(103)^a^	(99)^a^			
HbA1c trimester 3, mmol/mol	42 ± 10	39 ± 11	1.037	1.004–10.71	0.026
	(80)^a^	(77)^a^			

T1DM, type 1 diabetes mellitus; LGA, large for gestational age; BMI, body mass index; OR, odds ratio; CI, confidence interval.

Results are given as n (%) or mean ± SD.

^a^Number of samples available.

Variables marked with bold italics were significant in the final multivariable model.

**Table 4:** Results of univariable logistic regression analysis of variables tested for associations with LGA in women with T2DM.

	LGA (n = 20)	Non-LGA (n = 67)	OR	95% CI	*p*-value
Age at delivery, years	34.2 ± 5.4	34.5 ± 4.8	0.974	0.877–1.083	0.630
Smokers	0	11 (16)	-	-	-
Primipara	3 (15)	13 (19)	0.656	0.187–2.881	0.656
Weight in early pregnancy, kg	96 ± 24	81 ± 18	1.038	1.010–1.066	0.006
BMI in early pregnancy, kg/m^2^	34.3 ± 6.8	31.0 ± 5.8	1.095	1.006–1.192	0.036
Gestational weight gain, kg	14 ± 7	9 ± 6	1.130	1.028–1.241	***0*.*011***
Excessive weight gain	12 (60)	20 (30)	3.525	1.250–9.937	*0*.*017*
HbA1c trimester 1, mmol/mol	45 ± 12	44 ± 13	1.006	0.960–1.053	0.810
	(14)^a^	(55)^a^			
HbA1c trimester 2, mmol/mol	39 ± 9	35 ± 10	1.036	0.977–1.099	0.240
	(17)^a^	(56)^a^			
HbA1c trimester 3, mmol/mol	36 ± 7	36 ± 8	1.013	0.942–1.089	0.722
	(16)^a^	(46)^a^			

T2DM, type 1 diabetes mellitus; LGA, large for gestational-age; BMI, body mass index; OR, odds ratio; CI, confidence interval.

Results are given as n (%) or mean ± SD.

^a^Number of samples available.

The variable marked with bold italics was significant in the final multivariable model.

Multivariable logistic regression analysis was used to determine the independent risk of variables tested for an association with LGA in univariable analysis. All variables were included in the respective model with the exception of third-trimester HbA1c in T1DM (due to a large number of missing values) and HbA1c (all trimesters) in T2DM (due to an overall large number of missing samples). The multivariable model identified GWG (OR = 1.107, 95% CI: 1.044–1.17, *p* = 0.001) and second-trimester HbA1c (OR = 1.047, 95% CI: 1.015–1.080, *p* = 0.004) as independent risk factors for LGA in women with T1DM. GWG was also identified as an independent risk factor for LGA in women with T2DM (OR = 1.175, 95% CI: 1.048–1.318, *p* = 0.006), whereas BMI in early pregnancy and excessive GWG were not found to be associated with LGA in T1DM or T2DM pregnancies.

## Discussion

In agreement with other studies, the proportion of offspring with fetal overgrowth from women with T1DM and T2DM was high [2, 10], and higher in T1DM pregnancies than in T2DM pregnancies. Irrespective of diabetes type, GWG was identified as a major risk factor for fetal overgrowth, as recently shown by others [11, 12]. Furthermore, glycemic control (expressed as HbA1c) was identified as a risk factor for LGA in women with T1DM, whereas no associations with LGA were found in women with T2DM.

HbA1c is a crude measure of glucose control and does not take postprandial changes in glucose into account, which are known to affect the growth of the fetus [18]. Interestingly, Damm et al. reported that plasma glucose > 11 mmol/L and per cent glucose values outside the normal range in the third trimester were significant predictors of LGA and macrosomia in T1DM women [19]. Unfortunately, we did not have access to individual glucose measurements in the present material. Previous studies have shown that higher HbA1c levels in the second and third trimesters relate to adverse outcomes of pregnancy in women with pre-existing T1DM and T2DM [20]. A prospective study of 725 women with T1DM showed significantly increased rates of LGA babies with a maternal HbA1c of ≥ 42 mmol/mol at 26 and 34 weeks of gestation [21]. Moreover, Glinianaia et al. found increasing third-trimester HbA1c to be a stronger predictor of increased birth weight than second-trimester HbA1c in women with T1DM and T2DM [1]. However, this has not been a universal finding in all studies, as some have shown a stronger effect of HbA1c obtained in early pregnancy rather than in late pregnancy [22–25]. Due to a large number of missing values, third-trimester HbA1c was not included in our prediction model, but in line with some of the previous studies we found that second-trimester HbA1c, but not first-trimester HbA1c, was an independent risk factor for LGA in women with T1DM.

Alongside the increasing prevalence, there has been an increasing interest in pregnancies complicated by T2DM in recent years. Despite the fact that they have a milder glycemic disturbance, women with T2DM have perinatal outcomes that are comparable to those of women with T1DM [26]. In line with this, Clausen et al. reported worse perinatal outcomes in women with T2DM than in those with T1DM and in the background population [27]. As with our findings, a higher proportion of women with T2DM were older, had higher BMI, were multiparous and were of non-Nordic origin, but had lower HbA1c levels than those with T1DM. It has been hypothesized that substrates other than glucose also have a role in the pathogenesis of fetal overgrowth, including insulin resistance, other components of the metabolic syndrome, and hypertriglyceridemia [28]. In the present study cohort, there were few T2DM women with LGA outcome (n = 20), and there were a rather large number of missing values for HbA1c, making it difficult to test for an association in the multivariable model. In the univariable analysis, however, HbA1c had no effect on the incidence of LGA in T2DM women.

Pre-term birth was more prevalent in T1DM women (29%) than in T2DM women (13%). Despite this, the mean birth weight of the offspring of T1DM women was higher. The rate of cesarean section followed the same trend (42% and 34%, respectively), which can be compared to an overall rate of 17.7% in Sweden [29]. Women with T1DM gave birth at a mean of 37 weeks, most often through the decision of the antenatal team to deliver by induction of labor or cesarean section when signs of maternal or fetal complications developed.

It is notable that a higher proportion of women with T1DM gave birth to LGA infants (50%) than women with T2DM (23%). They generally gained more weight during pregnancy also, exceeding the IOM guidelines in 51% of cases as compared to 37% in women with T2DM. The reason for this discrepancy is not known, but it has been shown that lean pregnant women accrue significantly more fat mass than obese women [30]. However, this finding has not been confirmed by others [11, 31]. Moreover, it is important to note that the significant association between GWG and LGA was unrelated to BMI in the present study.

The majority of the women with T1DM had achieved near-normal HbA1c levels in the second trimester. Such strict glycemic control is challenging to achieve in T1DM due to the increased risk of hypoglycemia. “Overeating” due to frequent episodes of hypoglycemia could possibly partially account for the increased weight gain in women with T1DM. Another contributory factor may be lowering of maternal glucose levels driven by the fetus through a process called the “fetoplacental glucose steal phenomenon”. Early establishment of fetal hyperinsulinemia will increase the glucose gradient across the placenta through its effect on lowering of fetal glycaemia, and consequently lead to an increased flow of glucose to the fetus [32]. Poor glycemic control early in pregnancy will result in the establishment of fetal hyperinsulinemia, causing an exaggerated uptake of glucose by the fetus.

Treatment with metformin may help reduce maternal weight gain in women with T2DM but it does not appear to have an effect on fetal growth [33]. Since only a small proportion of women with T2DM were treated with metformin, an effect of metformin on maternal weight gain and fetal growth seems less likely. However, the fact that a higher proportion of T2DM women had a mixed ethnic background may have influenced the results.

In this study, we could not confirm that BMI (pre-pregnancy or in early pregnancy) is an independent risk factor for LGA in women with T1DM or T2DM. This could be explained by differences in study group characteristics, including the fact that the women in the cohort were generally leaner than in some of the previous studies [10, 34, 35]. Excessive GWG was more common in women who gave birth to LGA offspring, but excessive GWG was not identified as a predictor of LGA in the corresponding multivariable model. Considering the IOM guidelines on GWG, our results suggest that the weight gain in itself is of greater importance for the risk of LGA than GWG exceeding the IOM recommendations. However, since the numbers of women in each BMI category became rather small when dividing GWG into the different BMI classes (normal weight, overweight and obese) there may have been a lack of statistical power when trying to establish whether there was significance. In this context, it is important to note that LGA was defined as birth weight above > 2 standard deviations of the expected birth weight according to the Swedish reference curve (approximately equivalent to the 97.5^th^ percentile), and not above the 90th percentile for the reference population, which is the most commonly used definition. This may have affected the results.

The present findings highlight the importance of counselling and of assisting diabetic women with dietary changes and changes in exercise during pregnancy [36]. Women should be advised about the IOM recommendations for GWG, and the risk of fetal overgrowth when GWG exceeds the recommendations. It has even been suggested that GWG within the lower range of the values recommended by the IOM might be appropriate for women with pre-gestational diabetes [13, 31]. A low-glycemic index diet during pregnancy aimed at avoiding excessive weight gain has been recommended [37, 38]. Leisure-time physical activity, defined as 30 minutes of moderate exercise on most days of the week, has been shown to reduce the risk of LGA [39]. Moreover, regular exercise (a cycling program) from the twentieth week of gestation until delivery has been associated with lower birth weight [40]. Whether or not these recommendations are also appropriate for women with pre-gestational diabetes should be investigated.

This study was limited by its retrospective nature and its sample size, with a rather large number of missing values for HbA1c. The strengths of the study were the inclusion of consecutive pregnancies from a geographically well-defined area and the fact that all the data were retrieved from electronic medical files and were therefore not affected by recall bias. Data were obtained from hospital scales, blood results, or patient record notes taken in the antenatal care unit/delivery ward. Furthermore, information on important confounders was available and was controlled for in the logistic regression models. It has previously been shown by our group that maternal characteristics such as age, parity, smoking, BMI, and pre-existing diabetes influence fetal growth during the last trimester of pregnancy [41].

## Conclusions

Higher GWG was found to be associated with higher birth weight of the offspring, independent of maternal BMI in women with T1DM and T2DM, and independent of maternal glycemic control in women with T1DM. Our findings suggest that one should concentrate more on weight gain in the clinical care of these women, to minimize the risk of fetal overgrowth in pregnancy.

## Supporting information

S1 File T1DMdata. Data-set.
(XLSX)
