# Correction: Fetal overgrowth in women with type 1 and type 2 diabetes mellitus

**DOI:** 10.1371/journal.pone.0310788

**Published:** 2024-09-17

**Authors:** Linnea Ladfors, Nael Shaat, Nana Wiberg, Anastasia Katsarou, Kerstin Berntorp, Karl Kristensen

The fourth author’s name is spelled incorrectly. The correct name is Anastasia Katsarou.

The correct citation is: Ladfors L, Shaat N, Wiberg N, Katsarou A, Berntorp K, Kristensen K (2017) Fetal overgrowth in women with type 1 and type 2 diabetes mellitus. PLoS ONE 12(11): e0187917. https://doi.org/10.1371/journal.pone.0187917.
